# Draft Genome Sequence of *Brucella abortus* S99: Designated Antigenic Smooth Reference Strain Used in Diagnostic Tests in India

**DOI:** 10.1128/genomeA.00824-14

**Published:** 2014-08-21

**Authors:** Rajeswari Shome, Natesan Krithiga, B. S. Padmashree, Jagadesan Sankarasubramanian, Udayakumar S. Vishnu, Jayavel Sridhar, Paramasamy Gunasekaran, Jeyaprakash Rajendhran, Habibur Rahman

**Affiliations:** a National Institute of Veterinary Epidemiology and Disease Informatics (NIVEDI), Hebbal, Bangalore, Karnataka, India; b Department of Genetics, School of Biological Sciences, Madurai Kamaraj University, Madurai, Tamil Nadu, India

## Abstract

*Brucella abortus* strain S99 is widely used for the preparation of colored, plain, recombinant and smooth lipopolysaccharide antigens for the preparation of *Brucella* diagnostic kits. The genome of this strain was sequenced and the length of the genome was 3,253,175 bp, with 57.2% G+C content. A total of 3,365 protein coding genes and 53 RNA genes were predicted.

## GENOME ANNOUNCEMENT

The members of genus *Brucella* are the etiological agents of brucellosis, one of the most common bacterial zoonoses. *Brucella* spp. infects a broad range of mammals and *B. abortus* preferentially infects cattle (1). Brucellosis has a great variety of clinical manifestations, making it difficult to diagnose. Therefore, the diagnosis of *Brucella* infections is confirmed by the isolation of *Brucella* or by the detection of an immune response to its antigens such as lipopolysaccharide (LPS). *B. abortus* S99 contains smooth LPS (S-LPS), which is the major antigenic and immunogenic structure on the surface of all smooth strains of *Brucella* (2). LPS is the major virulence factor of *Brucella* and LPS deficient strains have less virulence and intra-cellular survival potential. The surveillance and control of brucellosis have been vigorously initiated in a nationwide program by the Department of Animal Husbandry, Dairying and Fisheries (DADF), Ministry of Agriculture, Government of India and Department of Biotechnology, Government of India through its Network Project on brucellosis with 9 dedicated epidemiological centers throughout the country in 2011. The draft genome sequence and the annotation of the antigenic reference strain *B. abortus* S99 used for diagnostics are presented here.

We isolated genomic DNA from *B. abortus* strain S99 using a DNeasy kit (Qiagen, Hilden, Germany). The genome was sequenced using an Ion Torrent personal genome machine (Life Technologies, Carlsbad, CA). In total, 2,921,679 reads with an average read length of 213 bp were obtained, which yielded 624.46 Mb of total sequenced bases with 189-fold coverage. The *de novo* assembly was performed using MIRA version 3.9.17 (3) which yielded 25 contigs. The largest contig was 503,052 bp long. The draft genome sequence of the *B. abortus* strain S99 was 3,253,175 bp long, with 57.2% G+C content. The genome sequence was annotated using the RAST server (4) and NCBI Prokaryotic Genomes Annotation Pipeline (http://www.ncbi.nlm.nih.gov/genome/annotation_prok/process/). The rRNAs and tRNAs genes were predicted using RNAmmer (5) and tRNAscan-SE 1.21 (6) respectively. A total of 3,419 genes were predicted, of which 3,365 are protein-coding genes. Overall, 2,685 of the protein-coding genes were assigned putative functions and 680 genes were annotated as hypothetical proteins. A total of 53 RNA genes were predicted, of which 4 were rRNA and 49 were tRNA genes. RAST annotation showed that *B. abortus* bv.1 str. NI435a and *B. abortus* bv.1 str. NI474 are the closest neighbors of *B. abortus* S99.

Multilocus sequence analysis using nine genetic loci were used to identify the sequence type (ST) of this strain as described earlier (7). Based on the allelic profiles (2,1,1,2,1,3,1,1,1), *B. abortus* S99 was grouped to ST-1. Genes involved in the pathways responsible for virulence and defense mechanisms of the bacterium were identified. This includes genes responsible for antibiotic and toxic compound resistance, invasion, intracellular survival, etc. Further comparative genomic analysis with other *Brucella* genomes would be useful for the development of diagnostic tools for brucellosis.

### Nucleotide sequence accession numbers.

This whole-genome shotgun project has been deposited at DDBJ/EMBL/GenBank under the accession no. AWTU00000000. The version described in this paper is AWTU01000000.
